# Importing genetically altered animals: ensuring quality

**DOI:** 10.1007/s00335-021-09908-x

**Published:** 2021-09-18

**Authors:** M.-C. Birling, M. D. Fray, P. Kasparek, J. Kopkanova, M. Massimi, R. Matteoni, L. Montoliu, L. M. J. Nutter, M. Raspa, J. Rozman, E. J. Ryder, F. Scavizzi, V. Voikar, S. Wells, G. Pavlovic, L. Teboul

**Affiliations:** 1 PHENOMIN-Institut Clinique de la Souris, CELPHEDIA, CNRS, INSERM, Université de Strasbourg, Illkirch-Graffenstaden, 67404 Strasbourg, France; 2 The Mary Lyon Centre, Medical Research Council Harwell, Harwell Campus, Didcot, OX11 0RD Oxon UK; 3 Czech Centre for Phenogenomics, Institute of Molecular Genetics of the Czech Academy of Sciences, Vestec, Czech Republic; 4 Institute of Biochemistry and Cell Biology, Italian National Research Council (CNR), Monterotondo Scalo, Rome, Italy; 5 Department of Molecular and Cellular Biology, National Centre for Biotechnology (CNB-CSIC) Madrid and CIBERER-ISCIII, Madrid, Spain; 6 The Centre for Phenogenomics, The Hospital for Sick Children, Toronto, ON Canada; 7 Wellcome Sanger Institute, Wellcome Genome Campus, Hinxton, Cambridge, UK; 8 Neuroscience Center and Laboratory Animal Center, Helsinki Institute of Life Science (HiLIFE), University of Helsinki, Helsinki, Finland; 9 Present Address: LGC, Sport and Specialised Analytical Services, Fordham, UK

## Abstract

The reproducibility of research using laboratory animals requires reliable management of their quality, in particular of their genetics, health and environment, all of which contribute to their phenotypes. The point at which these biological materials are transferred between researchers is particularly sensitive, as it may result in a loss of integrity of the animals and/or their documentation. Here, we describe the various aspects of laboratory animal quality that should be confirmed when sharing rodent research models. We also discuss how repositories of biological materials support the scientific community to ensure the continuity of the quality of laboratory animals. Both the concept of quality and the role of repositories themselves extend to all exchanges of biological materials and all networks that support the sharing of these reagents.

## Comprehensive documentation for research reproducibility

More than 70% of scientists claimed in a Nature survey that they failed to reproduce another researcher’s experiments (Baker 2016). Many causes have been offered to explain the lack of reproducibility in biological research (van der Worp et al. 2010; Freedman et al. 2015). For research involving laboratory animals, a comprehensive validation of the genetic make-up of animal models was recognised as an essential prerequisite for research reproducibility (Justice and Dhillon 2016), although it does not guarantee the validity of a model for a specific research purpose.

Traceability and full documentation of materials have been identified as key aspects in enabling the validation of animal models and the reproducibility of research using them (Freedman et al. 2015). The ARRIVE guidelines provide biomedical science with a checklist of metadata that should be recorded and reported, including the biological and genetic details of animals used (Percie du Sert et al. 2020) and further guidance and systems are being developed (Bespalov et al. 2021). However, comprehensive documentation of these animals must also be preserved at the point at which biological materials are transferred between research groups, so that research models can be accurately described when next used. Unfortunately, sufficient and accurate information does not always accompany the transfer of biological materials. It is also critical that the quality of materials themselves is not compromised in the process of transfer between researchers. In this respect, a genotyping protocol unique to the specific genetic alteration of the line greatly contributes to ensuring the traceability of materials as it reduces the risk of animal/line misidentification (Bonaparte et al. 2013; Jacquot et al. 2019). However, this does not represent a full validation of the integrity of the mutation of interest, nor is it a definitive assurance of the genetic profile of animals, and even less of their overall quality.

## Aspects of genetically altered laboratory animal quality

Defining the quality of a laboratory animal is not simple. One operational definition of a “good quality” laboratory animal could be to answer the following two questions affirmatively: (1) Is the use of the initial genetically altered (GA) line appropriate for the scientific question asked? (2) Are the imported animals still appropriate to answer the question? Answering these questions requires a comprehensive description of several criteria, which are summarised in Table 1.

**Table 1:** Aspects of laboratory animal quality and potential consequences of quality variation

Criteria categories	Criteria	Consequences of poor quality
Allele of interest	Genomic alteration^a^	Phenotypes associated with the wrong genetic alteration
	Functional consequences of mutation	Misinterpretation of phenotypes
Contamination with other alleles	Animal mix-up or incomplete breeding records	Phenotypes may be due to the wrong genetic alteration
Genetic background	Additional mutations fixed in closed colonies, genetic drift	Phenotypes due to the wrong genetic alteration or background
	Animal mix-up or incomplete breeding records	Phenotypes erroneously associated with genetic background or alteration
Hosted in mice and other environmental factors	Microbiota	The microbiota may modulate the observed phenotype in animals
	Pathogens	Observed phenotype is erroneously associated with a genetic alteration instead of being recognised as a consequence of a pathogenic infection
	Nutrition, husbandry conditions	Environmental factors are modulating the phenotype

^a^See Table 2 for quality control checks. The allele of interest may have been poorly validated initially or mutated subsequently

Initial quality control usually focuses on the genomic alteration of interest, regardless of the technology used to generate the mutations (Burgio and Teboul 2020; Birling et al. 2021). Surprisingly, for most published models, the full sequence information of the altered allele is not publicly available or even deposited in a relevant database. This sequence information is often lost over time as research personnel move on. This lack of information impacts the ability of future users to fully validate the allele, and may ultimately lead to doubts as to the quality and reliability of the research derived from these animals. The materials available from the International Mouse Phenotyping Consortium are a good counterexample of this. The sequences for alleles generated by the Consortium are deposited to a central database that is accessible through the Consortium website (mousephenotype.org). In addition, detailed allele descriptions are deposited to MGI as well as to the repositories from which the mice are distributed. Upon importing a new model, all users should review the way in which the allele of interest was initially validated, taking into account how the genetic alteration was produced (techniques may include embryonic stem cells, additive transgenesis by pronuclear injection, or genome editing, discussed in Bunton-Stasyshyn et al. (2021), and in this commentary). The desirable criteria for complete allele validation are evolving over time as new technologies become available and are discussed in a dedicated section of this commentary. Complete validation of a genetic alteration also requires functional assessment of the altered allele and ultimately of genes surrounding the target locus (Gofflot et al. 2011; Maguire et al. 2014; West et al. 2016; Lindner et al. 2020, 2021).

Whilst validating the locus of interest is an aspect essential to the quality of animal models, it is equally critical to exclude the possibility that the imported individuals carry contaminating alleles. Indeed, imported animals may be issued from colonies in which genetic alterations have been combined, either intentionally, or through mix-ups between animals and/or erroneous genotyping. Some animals, within an otherwise correct export batch, may fail some quality criteria or may harbour additional genetic alterations. The carry-over of other engineered alleles must therefore be excluded in all individuals transferred. Robust practices in genotyping are, as ever, important in this context (Frendewey et al. 2010; Jacquot et al. 2019). Copy counting can be used to evaluate the number of alleles that bear cassettes commonly used in genome engineering, such as *neo* (Tesson et al. 2010), but this requires that all sources of potential genetic contamination be known. A more systematic screen for common genetic contaminants can be performed by employing a genotyping approach based on a modest-sized array that probes for common genetic elements such as Cre, loxP, neo and GFP (MiniMUGA; Sigmon et al. 2020). Also, it is important to perform an allele-specific assay for each model. For example, in the case of a Cre line importation, the use of a generic Cre assay is not sufficient, at least in the first instance. An allele-specific assay needs to be performed in order to make sure that the correct Cre line has been obtained. This again emphasizes the requirement of the allele sequence (Jacquot et al. 2019). Scientists with very little expertise in molecular biology may rely on commercial or institutional suppliers to secure support genotyping imported lines. These approaches again emphasize the need for the allele sequence.

The accuracy of the description of the genetic background is another essential criterion that should be validated when importing animal models, as this may also have a strong impact on the genotype–phenotype relationships (Sittig et al. 2016). This is a particularly challenging aspect to validate, as a full characterization—for example, by whole genome sequencing—is not a practical solution, at least for the time being. Some widely used genetic backgrounds only subtly differ from one another; however, such differences may have significant phenotypic consequences (as is the case, for example, with the various C57BL/6 lines and sublines; Zurita et al. 2011; Mattapallil et al. 2012; Simon et al. 2013; Åhlgren and Voikar 2019). In particular, a common instance in which changes in genetic background are unwittingly introduced is that of crossing with DNA recombinase- (Cre or Flp) expressing lines of uncontrolled origin. In this respect, good practices in animal colony management are the basis of genetic quality assurance as a means to identify unwanted outcrosses (Benavides et al. 2019). In particular, a comprehensive record of both genetic background and pedigree are useful to retrace the origin of human error or of spontaneous mutations that have become fixed by prior breeding practices. For retrospective assessment of genetic backgrounds, array-based genotyping approaches are a practical alternative to whole-genome sequencing with which to interrogate the genetic background of exchanged animals. They are available as a survey that employs probes that identify common inbred genetic backgrounds (MiniMUGA; Sigmon et al. 2020), or as a larger array of probes to interrogate a more complex genetic background (MUGA; Morgan et al. 2015).

The myriad of microorganisms that colonize the mouse—the microbiota—can have significant effects on animal model phenotypes (Franklin and Ericsson 2017). The microbiome composition of wild-type but also GA lines is determined by the environment in which the animals are housed (Parker et al. 2018; Montonye et al. 2018; Hansen and Hansen 2021). As a result, the same line in two different locations may yield markedly different experimental results and it is prudent to ascertain the reproducibility of the key expected phenotypes in a newly imported line (for a recent special issue on this theme, see Pavlovic et al. 2021). 16S rDNA sequencing is a simple method for assessing bacterial diversity between samples (Johnson et al. 2019) and can be obtained from many commercial sources if needed. The use of controlled flora (for example, the altered Schaedler flora) enables researchers to control the impact of the microbiome on phenotyping (Franklin and Ericsson 2017). However, the complexity of maintaining gnotobiotic colonies does not permit the application of this approach to a large number of animals or research models (Nicklas et al. 2015).

The health status (assessing viral, fungal, parasitic and bacterial agents) ascertains the microbiological quality of imported mice and identifies infections by known pathogens that may confound scientific results (FELASA working group on the revision of guidelines for health monitoring of rodents and rabbits et al. 2014). Even with a health status that meets specific criteria, transport of live animals comes with a risk of infection, and the absence of agents that affect phenotypes cannot be guaranteed. The presence of opportunistic agents or commensal organisms that modulate the phenotype in specific conditions, or of emerging or unknown or untested pathogenic agents, outbreaks, and/or false negative tests remain possibilities, unless excluded using caesarean delivery or embryo rederivation (Suzuki et al. 1996; Mahabir et al. 2008).

Finally, comprehensive documentation of the housing conditions from which GA animals are obtained (for example, husbandry, diet, or enrichment) represents important information to be disseminated when animals are transferred, as variations in phenotypic differences may result in imported GA lines (Sundberg and Schofield 2018) when these change.

## The known known

The starting point for information on animals to be imported is the published article that describes those animals. Details such as the expected genetic make-up, model validation and assays employed for colony genotyping should be described in the initial publication, but additional information is available from other online resources, including Mouse Genome Informatics (MGI) (Eppig, 2017) and repositories (see below).

However, the characterization may only have been sufficient for the initial use of the model, or indeed may not meet the current standard to consider the model satisfactorily validated. It is therefore essential to review which criteria were used for validation, how they were tested and what was the outcome. Classical examples of insufficiently validated GA models include Cre drivers that historically were checked for recombinase activity in the tissues or cell lineages of interest, but often not in other anatomical locations (Song et al. 2010; Wicksteed et al. 2010). In addition, new phenotypes may arise as these models are transferred onto other genetic backgrounds. An example is the RIP-Cre mouse line that expresses the Cre recombinase in pancreatic β-cells, displays glucose intolerance (Lee et al. 2006) and has alterations of pancreatic cell mass and islet number (Pomplun et al. 2007). However, this line does not exhibit altered glucose tolerance after backcrossing on C57BL/6 J (Fex et al. 2007). Even when the validation data are available and extensive, it is good practice to reproduce the characterization on receipt of stocks in case of a mix-up or drift in activity.

One important tool to ensure the traceability of materials is a good understanding and use of standard nomenclature (Montoliu and Whitelaw 2011; http://www.informatics.jax.org/mgihome/nomen/strains.shtml). The nomenclature of GA lines includes high-level information on the genetic backgrounds that were used to propagate the colonies. But it can also be useful to obtain additional information on how the colonies were maintained, to identify the potential for genetic drift; for example, it would be important to know whether animals were maintained as closed colonies as that is likely to introduce bias (Montoliu and Whitelaw 2018). The nomenclature of alleles is essential to document the origin of biological materials and support their traceability. However, the official nomenclature does not always convey by itself the full and complex description of the model (including the detailed description and sequence of the genetic alteration), nor does it guarantee the genetic make-up of the material it describes.

## Which criteria should be validated upon receipt of animals?

Genotyping errors and animal misidentifications do occur (Jacquot et al. 2019). Materials received by a public repository may not carry the mutation specified by the depositor (Lloyd et al. 2015). The actual genetic background also may be different from that which is anticipated, or may not have been fully documented. For example, many publications still refer to the C57BL/6 genetic background, whereas genetic and phenotypic differences between the C57BL/6J and C57BL/6N sub-strains are known to exist. Therefore, certainty about the received genetic alteration and genetic make-up may only be reached when sufficient validation criteria are reproduced from the newly imported materials.

Standard validation methods have evolved with genome-engineering technologies generally geared towards ascertaining two criteria: that the locus of interest bears the correct sequence and that additional copies of template DNA used in the process of genetic engineering were not integrated off-target. With classical gene-targeting methods, four standard criteria are used for allele validation (criteria and methods are detailed in Table 2; Ryder et al. 2013; Codner et al. 2020; Birling et al. 2021). GA animals generated by additive transgenesis are often only checked for the presence of the transgene. This overlooks the possibility of changes in copy number or transgene expression over time, both of which ought to be assessed at regular intervals through generations. The importance of also defining the locus of integration is beginning to be recognised (Cain-Hom et al. 2017; Goodwin et al. 2019). With genome editing, the validation strategy will depend on the type of genetic alteration (Mianné et al. 2017) but should be aimed at sequencing the locus of interest and ascertaining the copy number of donor or mobilised segments (Burgio and Teboul 2020). Genome editing in the embryo brings the additional challenge of working with mosaic founders, so the presence of the correct allele in founders does not guarantee the integrity of the resulting GA colony (Birling et al. 2017; Mianné et al. 2017; Codner et al. 2018).

**Table 2:** Validation of genetic alterations

Allele production	Validation criteria	Assay type
Classical gene targeting	Targeting the correct locus	Southern blot, loss-of-allele qPCR/ddPCR and/or long-range PCR
	Structure of the targeting cassette	PCR and sequencing of targeting cassettes
	Functionality of targeting cassette	PCR and sequencing of key functional sequences, functional assays
	Absence of extra copies	Southern blot, qPCR/ddPCR, plasmid backbone PCR
Additive transgenesis	Transgene	qPCR/ddPCR, Southern blot
		qPCR/ddPCR or expression study in subsequent generations
	Locus of insertion	Fluorescence in situ hybridisation, inverse PCR, targeted locus amplification, X-drop analysis
Genome-editing	Sequence of the region of interest	PCR and Sanger or next-generation sequencing
	Copy number of mobilised segments and donor sequences	ddPCR, qPCR, Southern blot

Upon importing a GA colony, it is important to check whether the genetic alteration was ascertained to a sufficient level during the process of model generation, as not all criteria may have been validated (Codner et al. 2018) and different assays afford different levels of confidence (Birling et al. 2017, 2021). Standard quality control methods can detect the majority of laboratory animal quality issues but they are not exhaustive and some impactful events remain difficult to detect (Sailer et al. 2021). In this respect new methods are continuously being developed that support a more comprehensive characterization of GA materials (Kulnane et al. 2002; Liang et al. 2008; de Vree et al. 2014; McCabe et al. 2019; Blondal et al. 2021).

Genetic validation is the minimum standard of validation. However, the increasing numbers of examples of unexpected functional outcomes of genetic modification suggest that a wider molecular characterization is desirable. Functional validation (Scekic-Zahirovic et al. 2016; Lindner et al. 2021) and/or checking for unexpected compensatory mechanisms (El-Brolosy et al. 2019) are not systematically performed when GA lines are created. It is prudent to consider these parameters, as they may ultimately invalidate a model that was proven to contain the correct genetic alteration. Important validation can also be obtained from more standard assays. Indeed, the detection of non-Mendelian distribution is a powerful and often underused method for identifying additional transgene integrations, particularly for lines obtained by additive transgenesis (Lindner et al. 2021) but also for any other type of mutagenesis (Montoliu 2012; Birling et al. 2017; Mianné et al. 2017; Codner et al. 2018).

Beyond validation of the locus of interest, an assessment of genetic background can be done using a large array-based genotyping approach as a practical alternative to whole-genome sequencing (MUGA; Morgan et al. 2015). Backcrossing imported GA animals with wild-type mice of reliable origin has the advantage of allowing the segregation of unexpected sequences (such as unwanted donor integrations with genome editing, unexpected engineered sequences, and/or spontaneous mutations) and is an opportunity to check Mendelian transmission of the mutation of interest. In addition, the health status of imported animals must be consistent with the current health status of the receiving facility. Transferring frozen materials (for example, GA embryos or sperm) can reset their microbiological status. Transporting mice from one animal facility to another impacts their microbiota and will result in significant changes in bacterial composition by five days post-arrival (Montonye et al. 2018). Co-housing with wild-type mice bred at the receiving facility or further breeding the GA mice for a few generations with such wild-type animals will result in equilibration of the microbiota (Moeller et al. 2018; Robertson et al. 2019).

An in-depth characterization of all of the aspects discussed in this commentary could represent a significant —and in some instances, prohibitive—investment, and may not be essential for all intended uses of the imported stock: for example, a precise knowledge of the genetic background, although most desirable to ensure full research reporting and reproducibility, may not be essential for data interpretation if control animals are littermates of the experimental cohort. Similarly, a varying microbiome will impact some, but not all biological parameters. Nevertheless, each of the aspects discussed here should at least have been considered in the process of planning and implementing the experimental design involving imported stocks.

## The role of repositories

Repositories play an essential role in preserving the quality of GA models, as archiving protects against accidental contamination and drift over time (Lloyd et al. 2015). They fulfil this function by preserving and distributing biological materials and ancillary information about the expected genetic make-up of the animals, the validation assays that were performed, and the genotyping assays employed for routine genotyping. In addition, repositories provide expert curation of standard nomenclature and genotype–phenotype annotation and can include a record of related scientific publications (see articles discussing repository resources in this issue).

Repositories are also a resource for the development (see for examples Scavizzi et al. 2015; Codner et al. 2020) and dissemination of knowledge relating to molecular assays for validation (see repositories’ websites). The repositories themselves can also be involved in extensive campaigns of genomic (Goodwin et al. 2019; Birling et al. 2021) or even functional validation of GA materials (for example, see https://www.jax.org/research-and-faculty/resources/cre-repository/characterized-cre-lines-jax-cre-resource).

In addition to biological materials, users can access a range of related information from repositories including nomenclature, description of genetic alteration and the details of genetic alteration validation, genotyping assay protocols and their results, pedigree information (where available), and, where sufficient resources exist, assistance with troubleshooting further molecular characterization. For this information to be comprehensive, repositories rely on the depositors to also share these data, when available, along with full allele sequence information when depositing biological materials.

Finally, although shipment of germplasm is preferred to avoid live animal transport, repositories can assist with the management of health status when shipping live mice. In addition, many repositories can facilitate access to GA animals of a given health status by offering the option of rederivation to facilities that cannot implement such processes.

## Conclusion

Reliable quality management and documentation of biological materials cover many aspects of genetics and environment that are essential for research reproducibility. Repositories of biological materials support the scientific community in ensuring the continuity of the quality of animal models used in research. We have described the concepts of quality management and the role of repositories in the context of rodent research models; however, these concepts and roles extend to all exchanges of biological materials and all networks that support the sharing of biological materials.
